# *Cetacean Morbillivirus*-Associated Pathology: Knowns and Unknowns

**DOI:** 10.3389/fmicb.2016.00112

**Published:** 2016-02-08

**Authors:** Giovanni Di Guardo, Sandro Mazzariol

**Affiliations:** ^1^Faculty of Veterinary Medicine, University of TeramoTeramo, Italy; ^2^Department of Comparative Biomedicine and Food Hygiene, University of PadovaPadova, Italy

**Keywords:** *Cetacean Morbillivirus*, pathology, pathogenesis, viral neuropathogenesis, immunohistochemistry

## Abstract

The present minireview deals with the pathology of *Cetacean Morbillivirus* (CeMV) infection in free-ranging cetaceans. In this respect, while “classical” CeMV-associated lesions were observed in the lung, brain, and lymphoid tissues from striped dolphins (*Stenella coeruleoalba*) and pilot whales (*Globicephala melas*) which were victims of the 1990–1992 and 2006–2008 epidemics in the Western Mediterranean, an apparent reduction in CeMV neurovirulence, along with a different viral antigen’s tissue and cell distribution, were found during the 2010–2011 and the 2013 outbreaks in the same area. Of remarkable concern are also the documented CeMV ability to induce maternally acquired infections in wild cetaceans, coupled with the progressively expanding geographic and host range of the virus in both Hemispheres, as well as in conjunction with the intriguing forms of “brain-only” morbilliviral infection increasingly reported in Mediterranean-striped dolphins. Future research in this area should address the virus-host interaction dynamics, with particular emphasis on the cell receptors specifying viral tissue tropism in relation to the different cetacean species and to their susceptibility to infection, as well as to the CeMV strains circulating worldwide.

Throughout the last 25–30 years, at least nine morbilliviral epidemics have affected free-ranging cetacean populations from different areas of the planet (Van Bressem et al., 2014). Striped dolphins (*Stenella coeruleoalba*) have been the main “target” of the four outbreaks occurred in the western Mediterranean between 1990 and 2013, with pilot whales (*Globicephala melas*) having been also involved in the 2006–2008 epidemic in the same area (Fernández et al., 2008; Raga et al., 2008; Di Guardo and Mazzariol, 2013; Casalone et al., 2014; Van Bressem et al., 2014). Bottlenose dolphins (*Tursiops truncatus*), at their time, have been the main species affected by the four outbreaks occurred, between 1982 and 2014, along the Atlantic seaboards of USA (1982, 1987–1988, 2013–2014) (Lipscomb et al., 1994b; Krafft et al., 1995; Di Guardo et al., 2005; Van Bressem et al., 2009, 2014) and Mexico (1994) (Lipscomb et al., 1994a, 1996; Krafft et al., 1995; Di Guardo et al., 2005; Van Bressem et al., 2009), while common dolphins (*Delphinus delphis ponticus*) were the species targeted by the 1994 epidemic in the Black Sea (Birkun et al., 1999; Di Guardo et al., 2005; Van Bressem et al., 2009).

*Cetacean Morbillivirus* (CeMV)-associated pathology mirrors that commonly seen in *Morbillivirus*-infected animal and human hosts (Kennedy, 1998; Di Guardo et al., 2005; Van Bressem et al., 2009, 2014; Morris et al., 2015). Indeed, a prominent and widespread lymphoid cell depletion is generally observed during CeMV infection, with affected cetaceans developing a severe immunodeficiency resulting, at its turn, in a number of secondary viral, bacterial, fungal, protozoan, and parasitic infections (Domingo et al., 1992; Di Guardo et al., 1995, 2005; Kennedy, 1998; Fernández et al., 2008; Van Bressem et al., 2009, 2014; Mazzariol et al., 2012). Some of the agents causing the aforementioned infections, such as *Herpesvirus* and *Toxoplasma gondii*, can also behave under given circumstances as primary neurotropic pathogens (Kennedy et al., 1992; Di Guardo et al., 2010, 2011b; Sierra et al., 2014a), with cerebral lesions in diseased animals resembling those found in CeMV-affected cetaceans (Domingo et al., 1992; Kennedy, 1998; Fernández et al., 2008; Raga et al., 2008; Van Bressem et al., 2009, 2014). CeMV-associated brain lesions are consistent with those of a viral encephalitis, whereas a subacute-to-chronic, broncho/bronchiolo-interstitial pneumonia frequently involves both lungs (Domingo et al., 1992; Di Guardo et al., 1995, 2005; Kennedy, 1998; Fernández et al., 2008; Raga et al., 2008; Van Bressem et al., 2009, 2014). Along with the viral inclusion bodies detectable in the cytoplasm and/or nucleus of infected cells, multinucleated syncytia - also called “*Warthin–Finkeldey*-type syncytia” when occurring at pulmonary level – may be considered the most peculiar pathological “hallmark” classically found in all the aforementioned tissues from CeMV-affected animals (Domingo et al., 1992; Di Guardo et al., 1995, 2005; Kennedy, 1998; Fernández et al., 2008; Raga et al., 2008; Van Bressem et al., 2009, 2014; Sierra et al., 2014b).

Clear-cut immunohistochemical (IHC) evidence of morbilliviral antigen is detectable in lymphoid tissues, lung, brain, and, to a lesser degree, in a variety of epithelial and glandular sites from infected cetaceans, with a strong immunolabeling being generally observed at the level of multinucleated syncytia and intracytoplasmic/intranuclear viral inclusions (Domingo et al., 1992; Di Guardo et al., 1995, 2005; Kennedy, 1998; Fernández et al., 2008; Raga et al., 2008; Van Bressem et al., 2009, 2014; Sierra et al., 2014b).

Of special concern, from the comparative neuropathology and viral neuropathogenesis perspective, are the “brain-only” forms of morbilliviral infection which have been increasingly reported, after the 1990–1992 and the 2006–2008 epidemics, among CeMV-affected striped dolphins in the western Mediterranean (Domingo et al., 1995; Di Guardo et al., 2011a, 2013; Soto et al., 2011), with two additional cases having been also described in a CeMV-infected bottlenose dolphin from the same area (Di Guardo et al., 2013) as well as in a wild, “hospitalized” white-beaked dolphin (*Lagenorhynchus albirostris*; Van Elk et al., 2014) and in four striped dolphins stranded along the Canary Islands’ coasts between 2002 and 2011 (Van Bressem et al., 2014). Animals suffering from this peculiar form of infection show evidence of morbilliviral genome and/or antigens exclusively in the brain, with their lesions closely resembling those found in *Measles Virus* (MeV)-infected, “subacute sclerosing panencephalitis” (SSPE)-affected patients (Sato et al., 2012; Di Guardo and Mazzariol, 2013). In this respect, provided that the host- and the virus-related factors driving host-CeMV interaction are largely undefined, special emphasis should be placed upon the mechanisms underlying viral colonization and persistence within the brain of chronically infected dolphins (Di Guardo et al., 2013). Indeed, the cell receptor accounting for morbilliviral lymphotropism (*Signalling Lymphocyte Activation Molecule*, SLAM, or CD150) is not expressed by neurons, which could also be true for nectin-4, another cell receptor involved in morbilliviral epitheliotropism (Sato et al., 2012). Despite this, nectin-4 has been recently suggested to be implicated in the neurovirulence of *Canine Distemper Virus* (CDV; Pratakpiriya et al., 2012), a well-recognized model for the comparative study of human and animal morbilliviral infections (Reuter and Schneider-Schaulies, 2010; Sato et al., 2012).

Among the many hitherto open issues, of particular relevance is that concerning the “strategies” utilized by morbilliviruses for colonizing and “hiding” themselves inside the brain parenchyma of their susceptible host species, thereby giving rise to MeV-induced SSPE in man (Reuter and Schneider-Schaulies, 2010; Sato et al., 2012) and to its “animal analogues/counterparts”, exemplified by CDV-infected, “old dog encephalitis”-affected dogs, as well as by CeMV-infected-striped dolphins (Domingo et al., 1995; Di Guardo et al., 2011a, 2013; Soto et al., 2011). Nevertheless, it must be also underlined that the “brain lesions’ phenotype” should not be regarded as the exclusive “parameter/criterion” on the basis of which these “brain-only” forms of morbilliviral infection in striped dolphins may be considered “reliable disease analogs/counterparts” of SSPE in human patients. As a matter of fact, while Mediterranean-striped dolphins, with special reference to adult animals, have been reported to be quite commonly affected by “post-epidemic, brain-only forms of CeMV infection” (Domingo et al., 1995; Di Guardo et al., 2011a, 2013; Soto et al., 2011), SSPE is a rare disease condition of children and young adults, involving 8 to 20 per 1 million of MeV-infected individuals (Garg, 2008; Oldstone, 2009; Kweder et al., 2015). Additionally, and even more important, the “wild type” MeV strains/genotypes responsible for SSPE cases in man have been reported to carry several mutations, mainly affecting the matrix protein (M) gene and, less frequently, also the fusion protein (F) and the haemagglutinin (H) genes, with such mutations having been linked to increased viral spread as well as to an impaired ability of MeV strains/genotypes to produce cell-free, infectious viral particles (Garg, 2008; Oldstone, 2009; Moulin et al., 2011; Kweder et al., 2015).

To the best of our knowledge, no similar studies have been hitherto conducted on striped dolphins affected by “brain-only” forms of morbilliviral infection. Investigations of this kind would be of crucial relevance in order to definitely establish, among others, “to what extent” striped dolphins with “brain-only” forms of CeMV infection are “reliable models” for the comparative study of SSPE-affected humans.

Of additional concern are also the cell populations, both neuronal and non-neuronal, which are sequentially targeted following viral neuroinvasion. In this respect, it would be of interest to investigate whether given CeMV strains are characterized by a “selective/exclusive neurotropic behaviour” inside their hosts, a process which could be driven by the specific interaction of the viral pathogen with a receptor molecule expressed by neurons and/or other brain cells (Di Guardo and Mazzariol, 2013). During CDV infection, for example, the prolonged viral persistence inside the dog’s brain has been related to a non-cytolytic, astrocyte-to-astrocyte spread of the agent through a hitherto unidentified glial cell receptor (Wyss-Fluehmann et al., 2010; Alves et al., 2015).

A further issue of relevant concern is represented by the possibility that CeMV infection may be also acquired through “vertical” transmission, from the mother to the embryo/*fetus*/calf, in a similar manner to what reported for CDV infection in neonatal dog pups (Pandher et al., 2006).

Indeed, this has been suspected, if not even documented, in several CeMV-susceptible Odontocetes and Mystycetes, such as striped dolphins (Domingo et al., 1992; Di Guardo et al., 2011a), pilot whales (Fernández et al., 2008), sperm whales (*Physeter macrocephalus*; West et al., 2015), and fin whales (*Balaenoptera physalus*; Mazzariol et al., 2016). Such an elegant infection’s “strategy” could provide, among others, an agent “traditionally” characterized by a high virulence like CeMV with an additional “pathogenic potential”, which could be displayed by taking advantage of the reduction in immune response efficiency “physiologically” occurring during pregnancy (Sykes et al., 2012). In this respect, another research priority would be to investigate the immunopathogenesis of CeMV infection in T-helper (Th1)-dominant vs Th2-dominant, susceptible cetaceans (Van Bressem et al., 2014). Indeed, as the natural history and biology of *Human Immunodeficiency Virus* (HIV) – as well as of many other human and animal infection models – have taught, the infection’s course and outcome tend to be markedly different in Th1-dominant as compared to Th2-dominant patients, the population’s subset prone to develop full-blown AIDS (Romagnani, 2000).

Forthcoming studies in this area should be particularly focused, therefore, on the expression levels of SLAM/CD150 viral receptors in Th1-dominant vs Th2-dominant, CeMV-susceptible/infected cetacean hosts (as well as on the expression levels of additional virus receptors in different tissues). It would be also worthwhile to investigate the complex and largely unknown CeMV–host interaction dynamics, paying special attention to the synergistic role, if any, played by a number of immunotoxic and neurotoxic, persistent environmental pollutants, high concentrations of which are commonly found in tissues of “top predators” such as Odontocete cetaceans (Fossi et al., 2007).

Finally, two additional issues would deserve adequate consideration.

The first one regards the progressively expanding CeMV host range, as highlighted by the cases of infection recently documented in western Mediterranean fin whales (Di Guardo et al., 2011c; Mazzariol et al., 2012, 2016; Casalone et al., 2014) and, even more strikingly, also in a captive common seal (*Phoca vitulina*) hosted by an aquatic park near Rome (Mazzariol et al., 2013). While this fact could be partially biased by the increased number of studies carried out, in the near past, on wild cetaceans from different world regions, of remarkable concern are also the “emerging” CeMV strains recently identified in single, free-ranging cetaceans of different Odontocete species from Hawaii (West et al., 2013) as well as from the southern Hemisphere (Groch et al., 2014; Stephens et al., 2014). The genetic differences between the aforementioned isolates and those hitherto characterized from wild cetaceans of the northern Hemisphere (West et al., 2013; Groch et al., 2014; Stephens et al., 2014) are so prominent that it would be even questionable to include them inside the CeMV “clade” (Di Guardo and Mazzariol, 2014; Van Bressem et al., 2014). Furthermore, the “pathological phenotype” observed in CeMV-affected animals from the southern Hemisphere has been reported to diverge from that “classically” seen in infected cetaceans of the northern Hemisphere (Groch et al., 2014; Stephens et al., 2014). As a matter of fact, no evidence of morbilliviral genome and/or antigen was found within the brain parenchyma of the two Indo-Pacific bottlenose dolphins (*Tursiops aduncus*) in western Australia from which one of these “newly emerged” CeMV isolates was recovered (Stephens et al., 2014). Still noteworthy, although the CeMV strains identified in striped dolphins, pilot whales, fin whales and other free-ranging cetaceans during the four outbreaks occurred between 1990 and 2013 in the western Mediterranean appear to be genetically similar to each other (Fernández et al., 2008; Van Bressem et al., 2009, 2014; Bellière et al., 2011; Di Guardo et al., 2011c, 2013; Rubio-Guerri et al., 2013), the “pathological phenotype” characterizing the 2010–2011 and the 2013 outbreaks diverged from that of the 1990–1992 and the 2006–2008 epidemics in terms of viral tropism and pathogenicity, with special emphasis on neurotropism and neurovirulence, as well as in terms of lesions’ and viral antigen’s distribution (Rubio-Guerri et al., 2013; Casalone et al., 2014).

On the basis of what above, adequate research efforts would be absolutely needed on the cell receptors specifying the viral tissue tropism in relation to the different cetacean species and to their susceptibility to infection, as well as to the CeMV strains circulating worldwide.

## Author Contributions

GDG wrote the first draft of the minireview, which is the result of a “critical and prospective analysis” of the available literature, to which the two authors of the manuscript have also contributed throughout time. SM also carried out, at his turn, a “critical and prospective analysis” of the available literature, thereby carefully reviewing and integrating the initial paper’s draft, the last version of which was entirely agreed by the two authors. Finally, both GDG and SM made and shared in the last review’s version a number of comments, remarks, and suggestions on the “research priorities” to be addressed in the next future regarding the pathogenetic characterization of Cetacean Morbillivirus (CeMV) infection, with special emphasis on its neuro-immunopathogenesis (also in relation to its disease “counterparts” in man as well as in dogs).

## Conflict of Interest Statement

The authors declare that the research was conducted in the absence of any commercial or financial relationships that could be construed as a potential conflict of interest.
